# Conversation-based AI for anxiety disorders might lower the threshold for traditional medical assistance: a case report

**DOI:** 10.3389/fpubh.2024.1399702

**Published:** 2024-09-20

**Authors:** Martin Grosshans, Torsten Paul, Sebastian Karl Maximilian Fischer, Natalie Lotzmann, Hannah List, Christina Haag, Jochen Mutschler

**Affiliations:** ^1^Department of Global Health, Safety and Well-being, SAP SE, Walldorf, Germany; ^2^Psychiatric Services Lucerne, Lucerne, Switzerland; ^3^Institute of General Practice and Family Medicine, University Hospital of the Ludwig-Maximilians University of Munich, Munich, Germany; ^4^Institute for Implementation Science in Health Care, University of Zurich, Zurich, Switzerland; ^5^Epidemiology, Biostatistics and Prevention Institute, University of Zurich, Zurich, Switzerland

**Keywords:** anxiety, disorder, artificial intelligence, Chatbot, large langauge models

## Abstract

Artificial intelligence (AI) offers a wealth of opportunities for medicine, if we also bear in mind the risks associated with this technology. In recent years the potential future integration of AI with medicine has been the subject of much debate, although practical clinical experience of relevant cases is still largely absent. This case study examines a particular patient’s experience with different forms of care. Initially, the patient communicated with the conversation (chat) based AI (CAI) for self-treatment. However, over time she found herself increasingly drawn to a low-threshold internal company support system that is grounded in an existing, more traditional human-based care structure. This pattern of treatment May represent a useful addition to existing care structures, particularly for patients receptive to technology.

## 1 Introduction

Artificial intelligence (AI) offers a wealth of opportunities for medicine, if we also bear in mind the risks associated with this technology. The artificial intelligence program “Chatbot Generative Pre-trained Transformer (ChatGPT)” was released in November 2022 and made available to private users. ChatGPT is an AI chatbot based on Large Language Models (LLMs) that can communicate with users via text-based messages that mimic human language and are intended to provide relevant information during a conversation. This tool has quickly become a cultural phenomenon and is considered by many to be the next stage of digital support in human medicine.

The field of psychiatry and mental health still relies primarily on the doctor-patient relationship and on practitioner experience, with device-based medical findings currently playing a less important role, particularly regarding mild to moderate expressions of many psychiatric conditions. Against this backdrop, conversation (chat)-based AI (CAI) has the potential to offer new support in diagnosis and therapy.

In recent years the potential future integration of AI with medicine has been the subject of much debate (1–4), although practical clinical experience of relevant cases is still largely absent. Furthermore, as regards management of clinical cases, little progress has been made in the standardized, uniform incorporation of the newest generation of CAI within existing psychiatric-psychotherapeutic care structures.

This case study examines a particular patient’s experience with different forms of care. Initially, the patient communicated with the CAI for self-treatment. However, over time she found herself increasingly drawn to a low-threshold internal company support system that is grounded in an existing, more traditional human-based care structure. This pattern of treatment May represent a useful addition to existing care structures, particularly for patients receptive to technology.

To transform theoretical discussions into practical and manageable guidelines it is essential to first understand how to integrate CAI into traditional psychiatric care structures. Ideally, this new approach would supplement existing structures, leading to more efficient, accessible and lower-threshold psychiatric care. Studying relevant naturalistic examples is crucial to achieving this objective.

## 2 Case

We present a case study of a person who sought confirmation from psychiatric professionals regarding a partial remission of social phobia which she attributed to recommendations received through multiple ChatGPT interactions.

The patient gave written informed consent for the publication of this case report.

The patient reported experiencing social anxiety since adolescence but had self-managed her symptoms through extensive avoidance strategies without seeking medical diagnosis or treatment. If she was confronted with social situations she could not avoid, she experienced fear of being the center of attention. Furthermore, she experienced insecurity, sweating, tachycardia, mouth dryness, blush on the face and neck and trembling, hot flushes, feelings of apprehension, feelings of dizziness. Fulfilling the ICD-10 criteria for social phobia (ICD 10 40.1). All described symptoms were not present for the patient when the situation mentioned above were avoided. However, this behavior pattern was challenged when she transitioned to her professional life. Her work environment presented unavoidable social encounters, such as team meetings, discussions with colleagues and superiors, and giving presentations, which prompted her to seek medical attention. The patient experienced exacerbated symptoms during exposure, including severe anxiety, fear of blushing, trembling, sweating and tachycardia. These symptoms led in turn to anticipatory anxiety that occurred prior to social interactions, as well as a strong desire to avoid these situations.

The patient consulted her family doctor and was advised to seek outpatient psychotherapy. However, despite consulting three different psychotherapists, each therapy was discontinued after only a few sessions. The patient reported difficulty in establishing a therapeutic relationship with the therapists and experienced discomfort when discussing her symptoms, which she found shameful.

The patient, who has considered herself tech-savvy since adolescence, began using ChatGPT for psychoeducation and to manage her condition. She gained insight into the pathophysiology of social anxiety and learned about social anxiety disorder models. She also implemented therapeutic suggestions provided by ChatGPT, including relaxation exercises and mental and environmental exposure exercises related to anxiety-inducing social situations.

The patient reported that communicating with an anonymous chatbot at any time allowed her to express her fears and inhibitions without shame, resulting in significant relief. After several months of interacting with the CAI, she noted a reduction in her anxiety during social situations and a decrease in her physiological symptoms. The patient also highlighted the advantage of using CAI, as it avoided the social embarrassment she associated with face-to-face therapy sessions.

She found the CAI’s round-the-clock accessibility and quick responses particularly helpful in managing her condition. However, as her interactions continued, she began to question the validity and reliability of the AI’s advice. This was especially true when the software recommended consulting with a human mental health specialist in case of doubt.

To ensure peace of mind and safeguard her mental health during potential acute crises, the patient sought validation and a second opinion from a human expert, specifically a specialist in psychiatry and psychotherapy. Her company provided a virtual chat service with a psychiatric specialist, called ‘Ask the Expert’. The service was designed to be camera-free for easy, anonymous contact and to provide early-stage advice for patients with conditions such as social anxiety. The patient initially contacted the specialist via this tool, and due to her previous use of AI for self-treatment she felt less shame and was able to communicate more openly.

After reviewing her descriptions, we confirmed that the psychoeducation provided by the AI aligns with current medical knowledge about her condition, and that the suggested exercises and interventions are sound. It is plausible that interactions with the AI contributed to stabilizing and reducing her symptoms while also forming a possible surrogate ‘Doctor-Patient Relationship.’ It seems likely that this played a significant role in her decision to contact us after her symptoms stabilized.

To promote ongoing improvement and stabilization of her social anxiety, we recommended real-life, guideline-based measures, including group psychotherapy and regular follow ups with us, until now we had 2 meetings in 6 months in which we also informed the patient that further therapeutic measures such as pharmacotherapy would be available in the event of an exacerbation. The patient agreed to this approach while expressing a desire to continue interacting with the AI. We supported this dual-strategy approach, although we cautioned her not to follow the AI’s recommendations unquestioningly due to potential inaccuracies. We also recommended seeking expert advice in case of doubt.

## 3 Discussion

CAI will undoubtedly have a significant impact on medicine, especially in the fields of diagnosis and therapy in psychiatry and will likely play a significant role in conversational and counseling medicine, complementing existing standards (2, 5, 6). However, the development of CAI presents both opportunities and risks in its current form is certainly not suitable for all psychiatric diagnoses, especially not for acute conditions, which include many unresolved questions related to ensuring beneficial, safe and high-quality use of CAI.

One important question is how to standardize and structure the interaction between CAI-based programs and human specialists to ensure maximum safety and efficiency. Widespread practical, naturalistic clinical experiences with CAI or controlled studies that move beyond theoretical considerations are both currently lacking (4).

This case study raises the issue of the still insufficient integration and application of CAI in traditional medical infrastructure. In the case presented, a serendipitous event led to a successful dual strategy for this particular patient that combined the use of CAI with outpatient group psychotherapy and regular board-certified specialist consultations. This was made possible by proactive CAI interventions that enabled the patient to accept the offer of a company medical contact. Without this pre-treatment, the patient believes that she would have found even the low-threshold ‘Ask the Expert’ program too challenging.

It is worth noting that the patient explicitly confirmed that she would continue to use CAI as part of the treatment combination, thereby establishing a kind of therapeutic relationship with the system. As the suggested interventions have helped her in difficult situations in the past, she trusts the CAI while being fully conscious that these are interactions with a machine.

Although no clear data are available on the number of patients who independently use CAI to address clinical symptoms, we suggest that it is likely to be substantial. At this point we can only speculate on the numbers involved, the constellations of symptoms, the possible permanent avoidance of contacts with traditional psychiatric-psychotherapeutic structures, and whether patients are reluctant or willing to report CAI self-therapy. Although CAI systems advise consulting human specialists regarding mental health issues, patients May nevertheless underestimate the significant risks that accompany sole reliance on CAI. For example, the accuracy of recommendations is not verified by professional oversight. In serious cases, such as severe anxiety disorders or medical emergencies like suicidality that require immediate action, relying exclusively on CAI can be dangerous. Since no current CAI is capable of making these risk assessments, the authors strongly recommend that human specialists monitor the situation if possible. Furthermore, it is important to recognize that tools like ChatGPT and similar AI technologies are no substitute for psychotherapy. CAI systems are based on LLMs, which are capable of generating sophisticated text by learning from large data sets and identifying word relationships based on their training data (7). Clearly, AI models have no moral sense, empathy or understanding of human emotions, are incapable of experiencing compassion, sympathy, trust or respect, and therefore cannot truly understand a patient’s concerns. As demonstrated in this case study, CAI nonetheless holds promise both as a complement to mental health care and as a source of low-threshold individual digital support.

When using LLMs for medical queries, it is essential to conduct a careful risk–benefit analysis. Although LLMs can provide access to health information and potentially reduce or bridge waiting times for psychotherapy (8), they are not without significant risk. LLM-generated answers, even if they seem plausible, May contain serious errors or be entirely incorrect. Another important issue is data protection, since LLMs are often trained on user inputs that May include sensitive data provided without informed consent.

A major challenge when using CAI for mental health is maintaining strict privacy standards. The transmission of sensitive speech data, especially over public platforms such as ChatGPT, increases the risk of misuse and data breaches. While the advent of LLMs has transformed the field with efficient, accurate speech transcription and AI interaction, it also raises security concerns. In contrast to ChatGPT, a wide range of powerful LLMs are available open source (e.g., via the AI platform ‘Hugging Face’),^1^ making them well suited for deployment in secure IT environments and subsequent fine-tuning. Therefore, future efforts to integrate open-source LLMs into mental health care should focus on developing scalable, highly secure solutions that adhere to strict privacy and security protocols. Moreover, potential misuse of LLMs to spread misinformation in a health context is a serious concern and must be avoided. Regulating LLMs to ensure adherence to principles such as transparency, data protection, expert supervision and stringent content quality assessment is a topic worthy of further discussion. This May require standardizing CAI integration with existing medical infrastructures, which would necessitate a paradigm shift within traditional medical realms, a paradigm shift that May require concessions regarding initial treatment expertise while maintaining human treatment control (4).

Case reports are not systematic studies, but they can certainly lead to new ideas, approaches and ultimately to controlled studies that then examine the discovered by chance findings of case reports in a more systematic way. Further systematic research is necessary to support the integration of CAI in psychiatric-psychotherapeutic medicine. Therefore developing and training LLM models specifically for medical use (rather than relying on unregulated publicly available ChatGPT) should be developed in specialized centers to establish a regulated approval process for use in mental health by experts. As a consequence this future “medical AI” would be far more reliable in diagnostic-and treatment-related processes. Thus, the case study described above underscores the need for more research in this particular medical area.

## Data Availability

The original contributions presented in the study are included in the article/supplementary material, further inquiries can be directed to the corresponding author.

## Footnotes

^1^https://huggingface.co/
